# Supplementary material for: A Search for Snail-Related Answers to Explain Differences in Response of Schistosoma mansoni to Praziquantel Treatment among Responding and Persistent Hotspot Villages along the Kenyan Shore of Lake Victoria
Source: Am J Trop Med Hyg. 2019 Jun 3;101(1):65–77. doi: 10.4269/ajtmh.19-0089 (PMC6609173; doi:10.4269/ajtmh.19-0089)
Supplement: Supplementary file 1 [file tpmd190089.SD1.pdf]

Supplementary Table 1: Number of *B. sudanica* snails collected from each village and number of snails infected with *S. mansoni* or other trematode cercariae. SM = *Schistosoma mansoni*, ECH = echinostomes, AMPH = amphistomes, STRIG = strigeids, XIPH = xiphidiocercariae, SM:ECH = ratio of *S. mansoni* to echinostomes.

**VILLAGE**	Total Snails	SM	ECH	AMPH	STRIG	XIPH	Total Number of Infected Snails	Trematode Prevalence
				**Persistent Hotspot Villages**				
MINYA	1280	1	5	0	0	19	25	2.0
AGOK	429	7	4	0	1	7	19	4.4
MIGIRO	860	1	5	0	1	6	13	1.5
MIYANDHE	151	0	1	0	1	0	2	1.3
KANYIBOK	1304	28	22	7	4	1	62	4.8
USENGE	1322	23	10	6	12	14	65	4.9
**TOTAL**	5346	60	47	13	19	47	186	3.5
**%**		32	25	7	10	25		
				**Responding Villages**				
KOTIENO	1323	18	29	7	17	2	73	5.5
SEKA DOK	1296	3	10	2	4	1	20	1.5
WETA	1319	0	13	1	6	15	35	2.7
MUMBO	965	2	13	1	2	4	22	2.3
**TOTAL**	4903	23	65	11	29	22	150	3.1
**%**		15	43	7	19	15		
